# Physicochemical Characterization, Cytotoxicity, and In Vivo Evaluation of a Hydroxyapatite–Silver Composite for Bone Regeneration

**DOI:** 10.1155/bmri/8065442

**Published:** 2026-06-04

**Authors:** Agnes Andrade Martins, Aurigena Antunes de Araújo, Susana Barbosa Ribeiro, Maria Luiza Diniz de Sousa Lopes, Rômulo Camilo de Oliveira Melo, Valkleidson Santos de Araújo, Salomé Ribeiro da Silva, Renata Ferreira de Carvalho Leitão, José Sandro Pereira da Silva, Mauricio Bomio, Fabiana Villela da Motta, Mario Andrean Macedo Castro, Davi Neto de Araújo Silva, Flavia Q. Pirih, Marcos Vinicius de Carvalho Sousa Rosado, Carlos Augusto Galvão Barboza, Ruthineia Diógenes Alves Uchôa Lins

**Affiliations:** ^1^ Department of Dentistry, Federal University of Rio Grande Norte, Natal, Rio Grande do Norte, Brazil; ^2^ Department of Biophysics and Pharmacology, Federal University of Rio Grande Norte, Natal, Rio Grande do Norte, Brazil; ^3^ College of Pharmacy, Federal University of Rio Grande Norte, Natal, Rio Grande do Norte, Brazil; ^4^ Department of Morphology, Federal University of Ceará, Fortaleza, Ceará,, Estado, Brazil, ufc.br; ^5^ Department of Material′s Engineer, Federal University of Rio Grande Norte, Natal, Rio Grande do Norte, Brazil; ^6^ School of Dentistry, UCLA, Los Angeles, California, USA, ucla.edu; ^7^ Department of Morphology, Federal University of Rio Grande Norte, Natal, Rio Grande do Norte, Brazil

**Keywords:** biomaterial, bone regeneration, hydroxyapatite, inflammation, silver

## Abstract

This study is aimed at characterizing the physicochemical and biological properties of a hydroxyapatite–silver (HAp‐Ag) composite for bone regeneration. HAp and HAp‐Ag powders were analyzed using field emission scanning electron microscopy, energy‐dispersive X‐ray spectroscopy, X‐ray diffraction and fluorescence, and Fourier transform infrared spectroscopy. Cylindrical pellets obtained by powder pressing and sintering were evaluated for fracture toughness, compressive strength, relative density, porosity, and wettability. Cell viability was assessed using the Alamar Blue assay. In the *in vivo* experiment, an 8‐mm critical‐size calvarial defect was surgically created in 36 male Wistar rats randomly divided into three groups: control group (CG), untreated defect; HAp group, defect filled with HAp pellet; and HAp‐Ag group, defect filled with HAp‐Ag pellet. Euthanasia was performed after 90 days, and calvaria samples were analyzed by *μ*‐CT, histology, cytokine quantification, and gene expression analysis. The powders showed an irregular, agglomerated morphology, with HAp elements and silver detected in the HAp‐Ag sample. Incorporation of Ag into HAp increased fracture toughness (0.66–0.94 MPa.m^1/2^) and porosity (16.43%–25.95%) and reduced compressive strength (43.17–23.99 MPa) and contact angle (36.32°–10.51°). MC3T3‐E1 preosteoblastic cell viability was higher on HAp‐Ag surface compared to HAp pellets at Days 1 and 7. Compared to CG, defects filled with biomaterials showed greater bone formation and better bone quality parameters. In addition, a more advanced stage of bone maturation was observed in the animals treated with HAp‐Ag. HAp‐Ag composite upregulated TNF‐*α* and IL‐1*β* levels and Procollagen 3 and eNOS gene expression compared to CG. Silver incorporation into HAp increased fracture toughness and porosity, reduced compressive strength, and improved wettability. Despite these changes, HAp‐Ag enhanced cell viability and *in vivo* bone maturation, indicating that Ag addition does not compromise and may improve its bone regeneration potential.

## 1. Introduction

Bone plays essential roles in the human body, especially those related to locomotion, mechanical support, protection, and mineral homeostasis [1]. From a physiological point of view, bone tissue is under constant remodeling, a process that involves the reabsorption and formation of the extracellular bone matrix [2]. This mechanism allows it to adapt to the most diverse mechanical and biological stimuli to which it is constantly subjected [3, 4].

As an active multifunctional tissue, bones are susceptible to injuries, which, in addition to compromising their function, may reflect a compromised healing process and negatively impact the individual′s quality of life. [5–8]. In order to avoid this situation, regeneration strategies using grafts are constantly adopted in the areas of medicine and dentistry [9–12]. However, the repair of large defects, especially in the craniofacial region, resulting from congenital diseases, trauma, or surgeries, still presents a major challenge nowadays [13].

Autogenous bone is considered the “gold standard” material in the context of bone regeneration [14]. It is the only type of graft that presents the three properties involved in the process of bone neoformation: osteoinduction, osteoconduction, and osteogenicity [15]. Despite this, multiple disadvantages restrict its use, such as limited availability, the risk of vascular–nervous injuries and infections, and the increased morbidity associated with the procedure due to the involvement of a second surgical area [16].

Therefore, ceramic materials are widely used, particularly hydroxyapatite (HAp), which has similarities with the inorganic matrix of natural bone and is biocompatible, bioactive, and osteoconductive [17]. However, due to its low mechanical strength, the use of HAp alone is limited, particularly in cases of extensive rehabilitation or in regions prone to mechanical stress [18]. In this sense, the development of composites based on the association of HAp with other materials that provide better mechanical characteristics to the final product has become an increasingly explored field of study to overcome this situation, combining the advantages and overcoming the unfavorable characteristics of each type of material [19].

Silver is a very abundant metal in South America, and although research into its potential for bone regeneration is relatively recent [20], its low cytotoxicity and promising antimicrobial, healing, and osteogenic properties are well established in the literature [21–25]. Furthermore, the incorporation of silver into ceramic materials, including HAp, may be associated with an improvement in mechanical properties, as well as an increase in the longevity of the intervention [25, 26]. However, despite the current literature on silver–ceramic composites, important gaps remain. Previous studies have predominantly focused on antimicrobial activity and general biocompatibility, with limited investigation into the combined physicochemical characterization and *in vivo* performance of these materials. Thus, the present study is aimed at characterizing the physicochemical properties of a new hydroxyapatite and silver (HAp‐Ag) composite and evaluating its cytotoxicity and biological properties regarding the potential for stimulating new bone formation in critical‐size calvarial defects.

## 2. Materials and Methods

### 2.1. Synthesis of Biomaterials

For the synthesis of HAp and HAp‐Ag powders, the following reagents were used: calcium hydroxide (Ca(OH)_2_) (98% purity, Synth), phosphoric acid (H_3_PO_4_) (85% purity, Vetec), silver nitrate (AgNO_3_) (99.0% purity, Synth), ammonium hydroxide (NH_4_OH) (29% purity, Synth), and distilled water. HAp and HAp‐Ag powders were obtained from precursor solutions of Ca(OH)_2_, AgNO_3_, and H_3_PO_4_. The Ca/P molar ratio was maintained at 1.67 [27] and pH adjusted to 10 with NH_4_OH solution. The formulations are summarized in Table 1.

**Table 1:** Chemical formulations of samples and stoichiometric amounts of each precursor.

Formulation	Ca(OH)_2_		Ag(NO)_3_			H_3_PO_4_	
	mMols	(g)	mMols	(g)	%	mMols	(mL)
HAp	89.86	6.658	—	—		53.90	3.300
HAp‐Ag	89.77	6.651	0.09	0.0152	0.1	53.90	3.300

Firstly, the AgNO_3_ solution was added to the Ca(OH)_2_ solution. Then, the H_3_PO_4_ solution was dripped into the Ca‐Ag solution. After the addition of the precursors, the resulting solution underwent an aging process at 80°C under reflux and magnetic stirring for 23 h. The same procedure was followed for the preparation of pure HAp, but without the addition of AgNO_3_. After the aging time, the obtained product was centrifuged with distilled water to remove possible impurities. Finally, the powder was dried at 100°C in an oven for 24 h.

The morphology of HAp and HAp‐Ag powders was analyzed by a field emission gun–scanning electron microscope (FEG‐SEM) (Supra 35 VP, Zeiss) equipped with energy dispersive spectroscopy (EDS) (XFlash, Bruker) for qualitative elemental analysis of the samples. Crystalline phases of the samples were analyzed by X‐ray diffraction (XRD) (XRD‐7000, Shimadzu), with CuK*α* radiation (15404 Å), 10°–50° scan, and step size of 0.02°s^−1^. Chemical analysis of the powders was performed by X‐ray fluorescence (XRF) (EDX‐720, Shimadzu) using a rhodium tube and silver analyzer crystals. The presence of functional groups was evaluated by Fourier transform infrared (FT‐IR) spectroscopy (FT‐IR Vertex 70, Bruker) in the range of 400–4000 cm^−1^.

The powders were then pressed into cylindrical tablets with dimensions of 1 × 8 mm in a uniaxial hydraulic press (P15200, Bovenau). Twenty test specimens were made for each formulation, under a compaction pressure of 74 MPa. Finally, the specimens were sintered in a conventional muffle furnace at 1200°C, with a heating rate of 10°C.min^−1^ for 4 h.

### 2.2. Characterization of the Physical Properties of Biomaterials

In order to characterize the biomaterials, we evaluated their mechanical properties, relative density, porosity, and wettability.

The mechanical properties of the pellets were measured from fracture toughness and compressive strength tests. For the fracture toughness test, Vickers microhardness was measured on a Pantec HVS‐1000 microhardness tester. Images of the indentations and cracks in the tested samples were obtained using a scanning electron microscope (SEM) (TM3000, Hitachi). Using the hardness and crack measurement data, the expression of Anstis et al. [28] was used to calculate the mechanical property. The compression strength test was performed on a ZwickRoell Model Z2.5 testing machine. After testing, the fractured surfaces were examined using FEG‐SEM (Zeiss Supra 35‐VP) to analyze grain boundaries and the presence of pores.

The relative density and porosity of the sintered pellets were obtained following the ASTM C 20‐00 standard [29].

The wettability behavior (hydrophilic/hydrophobic) was evaluated by measuring the contact angle formed by water droplets on the wafer surfaces using an optical tensiometer (Theta Lite, Attension) at a temperature of 22°C ± 1°C and relative humidity of 55% ± 3%. The images were captured for 15 s after the droplet was deposited on the wafer surface. The data represent the average of three measurements.

### 2.3. Cell Viability

For cytotoxicity assays, MC3T3‐E1 murine preosteoblastic cells (American Type Culture Collection [ATCC], United States) were cultured on cylindrical discs of both HAp and HAp‐Ag for up to 7 days. Cells were maintained in *α*‐MEM medium supplemented with 10% fetal bovine serum (FBS) and 1% antibiotic–antimycotic solution (containing 100 IU/mL penicillin, 100 *μ*g/mL streptomycin, and 0.25 *μ*g/mL Amphotericin B, all from Gibco, United States). The culture medium was refreshed on Days 1, 3, and 5. Cell viability was assessed using the Alamar Blue assay (Invitrogen, United States) on Days 1, 3, and 7. Cells were seeded onto the surface of the discs in 24‐well plates at a density of 1 × 10^4^ cells/well. Cells cultured directly on the polystyrene surface of standard tissue culture plates were used as the positive control for cell growth, as this substrate is considered the gold standard for in vitro cell adhesion and proliferation. At each time point, the medium was removed, and cells were incubated with 10 *μ*L of Alamar Blue reagent diluted in 90 *μ*L of *α*‐MEM under standard conditions. After 4 h of incubation, absorbance was measured at 570 nm (reduced form) and 600 nm (oxidized form) using a microplate reader (Epoch, United States). The percentage of Alamar Blue reduction was calculated according to the manufacturer′s instructions and used as a measure of metabolic activity at each experimental time point. For comparative purposes, an additional analysis was performed in which the values obtained for each group were normalized to the control condition (cells cultured on standard tissue culture polystyrene), which was set at 100% at each time point. All the experiments were performed in octuplicate (*n* = 8).

### 2.4. *In Vivo* Study

The experimental protocol of this study was submitted to the Ethics Committee for Teaching and Research on Animals (CEUA)–Federal University of Rio Grande do Norte (UFRN) and approved under protocol number 019/2020. The authors followed the ARRIVE (Animal Research: Reporting of *In Vivo* Experiments) guidelines.

#### 2.4.1. Study Groups

The sample of the present study consisted of 36 male Wistar rats (*Rattus norvergicus*) provided by the Central Animal Facility of the Biosciences Center of UFRN, with approximately 250–350 g of body mass and an age of 60 days. During the acclimatization period (4 weeks) and the experimental period, the animals were kept in environmental conditions of humidity (45%–55%) and temperature (22°C ± 2°C) controlled with a standard light/dark cycle of 12/12 h, with water and chow for laboratory animals (Purina, Nuvital, SP, Brazil) ad libitum. Simple randomization was performed, and group allocation was conducted by a researcher not involved in outcome assessment. Thus, the animals were distributed into the following groups (n = 12 animals/group):

• Control group (CG): animals undergoing surgery for a critical calvarial defect, without treatment.
• HAp group: animals undergoing surgery for a critical calvarial defect, followed by placement of HAp pellets to fill the defect.
• HAp‐Ag group: animals undergoing surgery for a critical calvarial defect, followed by placement of HAp‐Ag pellets to fill the defect.

Sample size was based on previous experimental data from our research group using the same animal model and similar investigated outcomes [30–32]. Previous data demonstrated marked differences between groups in tumor necrosis factor‐*α* (TNF‐*α*) levels and in the relative gene expression of Bone Morphogenetic Protein 2 (BMP‐2), Runt‐Related Transcription Factor 2 (RUNX2), and Interleukin 6 (IL‐6), indicating large expected effect sizes (estimated Cohen’s *d* > 3). Considering these effect sizes, a significance level of 5% (*α* = 0.05), and a statistical power of 80%, four animals per group were considered sufficient to detect statistically and biologically relevant differences. Since the analyses were performed independently, the total number of animals corresponded to 12 per group across the entire study.

#### 2.4.2. Critical‐Size Defect in Calvaria

Initially, the animals were anesthetized by intraperitoneal (i.p.) injection of 10% ketamine hydrochloride (80 mg/kg, Vetnil, São Paulo, Brazil) and 2% xylazine hydrochloride (10 mg/kg, Calmium, São Paulo, Brazil). The surgical area was then shaved, followed by an incision with a scalpel (Blade 15) in the skin over the parietal bone to expose the calvaria. Using an 8‐mm diameter trephine drill attached to a contra‐angle and a dental micromotor (Kavo) and driven by a surgical motor (Driller), a symmetrical 8‐mm diameter defect was created under constant irrigation with saline solution to prevent overheating of the bone tissue. For the experimental groups (HAp and HAp‐Ag), after removal of the calvarial bone, the composites, previously sterilized by moist heat (121°C for 30 min), were delicately positioned in the defect. Finally, the periosteum and soft tissues were sutured with 4.0 nylon thread (Polysuture, São Paulo, Brazil). At the end of the surgical procedure, the animals received an i.p. dose of tramadol (5 mg/kg) and were wrapped in a heated blanket and observed throughout the anesthetic recovery period. Two animals from the CG were lost during the surgical procedure due to anesthesia‐related complications.

#### 2.4.3. Postoperative Follow‐Up

The animals were observed daily throughout the experimental period to assess soft tissue healing and well‐being. In the first five postoperative days, tramadol at a concentration of 0.1 mg/mL was added to the animals’ water. Moreover, during the first week after surgery, a 1% chlorhexidine digluconate solution (Needs, São Paulo, Brazil) was delicately applied to the suture region with the aid of sterile gauze.

#### 2.4.4. Euthanasia

After 90 days of the surgical procedure, the animals were euthanized by i.p. administration of a lethal anesthetic dose of 10% ketamine hydrochloride (240 mg/kg, Vetnil, São Paulo, Brazil) and 2% xylazine hydrochloride (30 mg/kg, Calmium, São Paulo, Brazil). Then, the skull region was carefully dissected, and samples from the defect region were used for the analyses described below.

#### 2.4.5. Micro‐Computed Tomography (*μ*‐CT)

Skull samples (*n* = 4 samples/group) were fixed with 10% buffered paraformaldehyde for 24 h and kept in 70% alcohol solution until analysis. The samples were then scanned using a high‐resolution *μ*‐CT (SkyScan 1275, Sky‐Scan N.V., Belgium) with an image resolution of 15 *μ*m, with a 55 kV, 167 *μ*A X‐ray source, and a 0.5‐mm aluminum filter. Then, the image datasets were reconstructed using the NRecon program (SkyScan N.V.) Version 1.7.4.6, with a ring artifact correction of 5, a beam hardness correction of 20%, and fine adjustment. After reconstruction, the images were viewed and reoriented in the transaxial, coronal, and sagittal planes with the DataViewer software (SkyScan N.V.).

Volumetric analysis was performed using CTAn software (SkyScan N.V.) Version 1.13.11.0. Regions of interest (ROIs) were standardized in 40 slices per sample, starting at the highest defect edge identified by the coronal view. Using the transaxial view, each selected slice received an individual delimitation with the same pattern, covering the entire defect area and extending 3 mm beyond the defect edge. Binary selection of a data set with automatic threshold values, morphometry, and 3D analysis was performed. Bone volume (BV) and tissue volume (TV) were measured to calculate percentage BV (BV/TV). In addition, the following parameters were evaluated: percentage porosity, trabecular separation, trabecular number, and trabecular thickness. All image processing and ROI selection procedures were performed by a blinded evaluator, who was unaware of the group allocation.

#### 2.4.6. Histological Analysis (Hematoxylin and Eosin [H&E])

Skull samples involving the defect region (*n* = 4 samples/group) were fixed with 10% buffered paraformaldehyde for 24 h. They were then decalcified in 10% ethylenediaminetetraacetic acid (EDTA) solution (pH 7.4) for 16 weeks. Subsequently, they were processed and embedded in paraffin blocks. The slides were obtained from 3.5‐*μ*m sagittal serial sections and subsequently stained with H&E. The ROI of the histological slides, equivalent to the margins of the bone defect, was analyzed blindly by a pathologist under a conventional optical microscope (OlimpusCH2, Olimpus Optical Co. Ltd., Japan), considering the following parameters:

▪ Stage of bone maturation [33]:
  o. Score 1: formation of connective tissue filling the defect with blood capillaries, fibroblasts, macrophages, and newly formed collagen fibers
  o. Score 2: presence of dense connective tissue, suggesting differentiation of bone tissue with a large number of osteogenic and osteoprogenitor cells with fibrous organization
  o. Score 3: presence of new bone in which the connective tissue differentiates to form or indicate bone matrix
  o. Score 4: formation of mature bone tissue
▪ Inflammation [31]:
  o. Score 0: absence of inflammatory cells
  o. Score 1: weak presence of inflammatory cells
  o. Score 2: moderate presence of inflammatory cells
  o. Score 3: intense presence of inflammatory cells

#### 2.4.7. Proinflammatory Cytokine Dosage (TNF‐*α*, IL‐6, and Interleukin 1*β* [IL‐1*β*])

Soft tissue samples (*n* = 4 samples/group) surrounding the graft region were removed and divided into two fragments (per animal), which were then used for cytokine analysis (frozen at −80°C for later analysis) and gene expression analysis (stored in TRIzol at −80°C for later analysis). For cytokine analysis, the samples were then homogenized and processed. Tissue levels of TNF‐*α*, IL‐6, and IL‐1*β* were obtained using commercial enzyme‐linked immunosorbent assay (ELISA) kits (R&D Systems, Minneapolis, Minnesota). Microtiter plates were coated overnight at 4°C with rat anti‐TNF‐*α*, anti‐IL‐6, and anti‐IL‐1*β* antibodies. The plates were then washed three times with wash buffer (0.05% Tween 20 in PBS, pH 7.2–7.4). The wells were blocked by adding 100 *μ*L of diluent reagent (1% BSA in PBS, pH 7.2–7.4) for 1 h at room temperature. The plates were washed three times with wash buffer. The samples and standards were then added for incubation for 2 h at room temperature. Immediately after, the plates were washed three times with wash buffer, and 100 *μ*L of the diluted detection antibody was added to each well for 2 h. After that, three washes were performed with wash buffer, and 100 *μ*L of streptavidin‐HRP (1:5000) was added to each well for 20 min at room temperature in the dark. Then, the last three washes with buffer were performed, with subsequent addition of 100 *μ*L of substrate solution to each well for 20 min at room temperature. The enzymatic reaction was stopped with 50 *μ*L of H_2_SO_4_, and measurements were performed in a spectrophotometer, with an absorbance of 450 nm.

#### 2.4.8. Reverse Transcription–Quantitative Polymerase Chain Reaction (RT‐qPCR)

For gene expression analysis, fragments stored in TRIzol at −80°C (*n* = 4 samples/group) were used. Total RNA (1 mg) was transcribed using the SV Total RNA Isolation System extraction kit (Promega Corporation, Wisconsin, United States). Cycling conditions were as follows: denaturation at 95°C for 5 min and then cycles of 95°C for 30 s, 60°C for 30 s, and 72°C for 30 s. Samples were cycled 40 times at 95°C for 30 s for cDNA denaturation, 60°C for 30 s, and cDNA annealing at 72°C for 30 s. The threshold cycle value for each reaction was recorded and analyzed using the Bio‐Rad IQ5Real‐Time Software Detection System, with the 2‐*ΔΔ*Ct method, used for relative quantification based on *β*‐actin as the reference gene. Primers were designed for the following genes: *β*‐actin (housekeeping gene), BMP‐2, RUNX2, Procollagen 1, Procollagen 3, transforming growth factor *β* (TGF‐*β*), Osterix, and endothelial nitric oxide synthase (eNOS), using the Primer Express software Version 3.0.1 (Applied Biosystems, United States). Forward and reverse primer sequences are detailed in Table S1.

### 2.5. Statistical Analysis

The data obtained were transferred to GraphPad Prism 8.01 software and subjected to normal distribution assessment using the Shapiro–Wilk test. The comparison between the HAp and HAp‐Ag groups in the cell viability assay was performed using an unpaired *t*‐test. Data from the *in vivo* experiments were statistically evaluated by analysis of variance (ANOVA) followed by Tukey′s posttest in case of normal distribution. In case of nonnormal distribution, the data were evaluated by the Kruskal–Wallis test followed by Dunn′s posttest. Statistically significant levels were set at 5% (*p* < 0.05).

## 3. Results

### 3.1. Characterization of HAp and HAp‐Ag Powders

**Figure 1:**
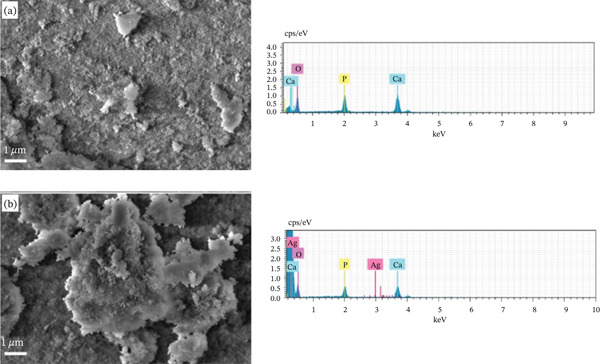
Scanning electron microscopy images for evaluation of powder morphology and their respective elemental analyses. (a) Hydroxyapatite (HAp) and (b) synthetic hydroxyapatite–silver (HAp‐Ag).

Figure 1 shows the FEG‐SEM images and the EDS spectrum of the obtained samples. According to the images, the powders presented irregular and agglomerated morphology. The elemental analysis of the samples was determined from EDS spectra. From the results, it was possible to confirm the HAp‐forming elements (Figure 1a) and the presence of silver in the doped sample (Figure 1b).

**Figure 2:**
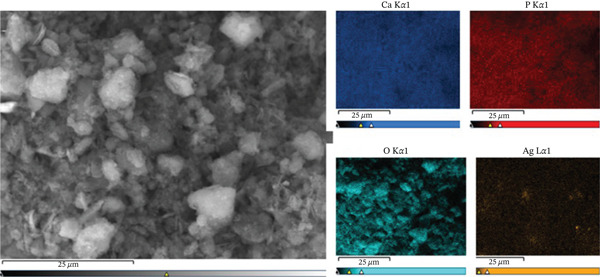
Elemental mapping of the synthetic hydroxyapatite–silver (HAp‐Ag).

Figure 2 presents the EDS elemental mapping of HAp‐Ag. As can be observed, there is a relatively even distribution of the element, in addition to the absence of micrometric agglomerates. This suggests that silver is not present as large metallic particles or macroscopic precipitates, but rather in the crystalline lattice of HAp.

**Figure 3:**
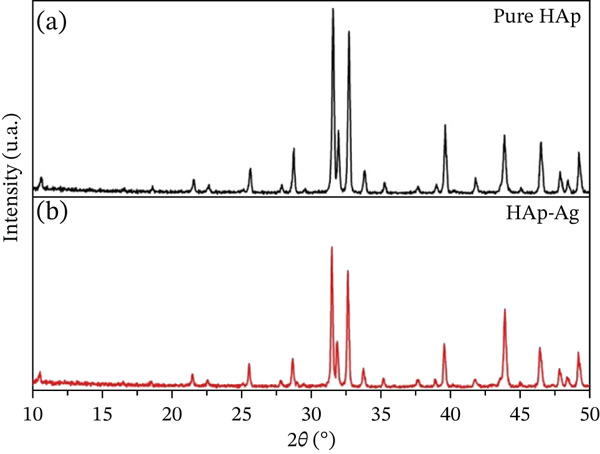
Relative intensity of the diffracted peaks in the characterization of the crystalline phases of the tablets of (a) synthetic hydroxyapatite (HAp) and (b) synthetic hydroxyapatite–silver (HAp‐Ag) by means of X‐ray diffraction.

Figure 3 shows the XRD results of the sintered HAp and HAp‐Ag samples. In the XRD pattern of HAp (Figure 3a), the formation of HAp with a hexagonal crystalline structure with space group P63/m was observed, without the presence of other phases, in accordance with ICDD‐PDF 9‐432 [34]. In the XRD pattern of HAp‐Ag (Figure 3b), there was no new phase, but a reduction in the intensity of the main peaks was observed. The magnification of the main peaks (Figure 3b) of the two samples reveals that doping with silver caused a shift of the diffraction peaks to smaller angles, which is commonly attributed to the expansion of the crystal lattice.

To confirm that there was a change in the crystal lattice, the crystallite size was determined using the Debye–Scherrer equation [35], and the microstrain was obtained using the Williamson–Hall equation [36]. Table 2 shows that after the substitution of calcium with silver, the crystallite size decreased, and the microstrain increased, confirming the changes in the crystal lattice indicated by the XRD spectra.

**Table 2:** Crystallite size and microdeformation of pure HAp and HAp doped with Ag.

Formulation	*L*(nm)	*ε*(10^−4^)
HAp	58.58	1.6
HAp‐Ag	56.2	1.67

Table 3 presents the results of the elemental chemical analysis of the powders by XRF. For HAp‐Ag, the silver content was 0.08%. Regarding the (Ca + Ag)/P ratio, the HAp and HAp‐Ag powders showed a value of 1.66 and 1.63, respectively.

**Table 3:** Results of chemical analysis by XRF and molar ratio (Ca + Ag)/P.

Formulation	Ca (%)	P (%)	Ag (%)	(*C* *a* + *A* *g*)/*P*
HAp	68.25	31.75	—	1.66
HAp‐Ag	67.81	32.11	0.08	1.63

**Figure 4:**
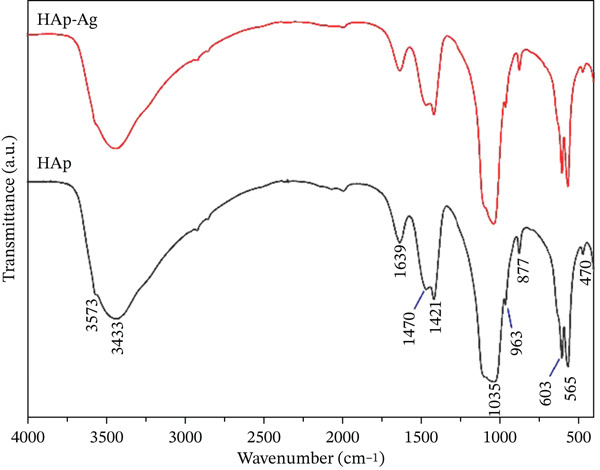
FT‐IR spectra of HAp and HAp‐Ag powder samples.

The spectra of FT‐IR spectroscopy are shown in Figure 4. Both samples exhibited an intense band at ~1639 cm^−1^ and broad bands in the 3400–3600 cm^−1^ region. The characteristic vibrational modes of PO_4_^3−^ were observed at ~470, 560–605, ~963, and ~1035 cm^−1^. A band at ~877 cm^−1^ and peaks in the 1400–1500 cm^−1^ region were also detected. No additional bands were observed in the HAp‐Ag sample.

### 3.2. Characterization of the Physical Properties of Biomaterials

**Figure 5:**
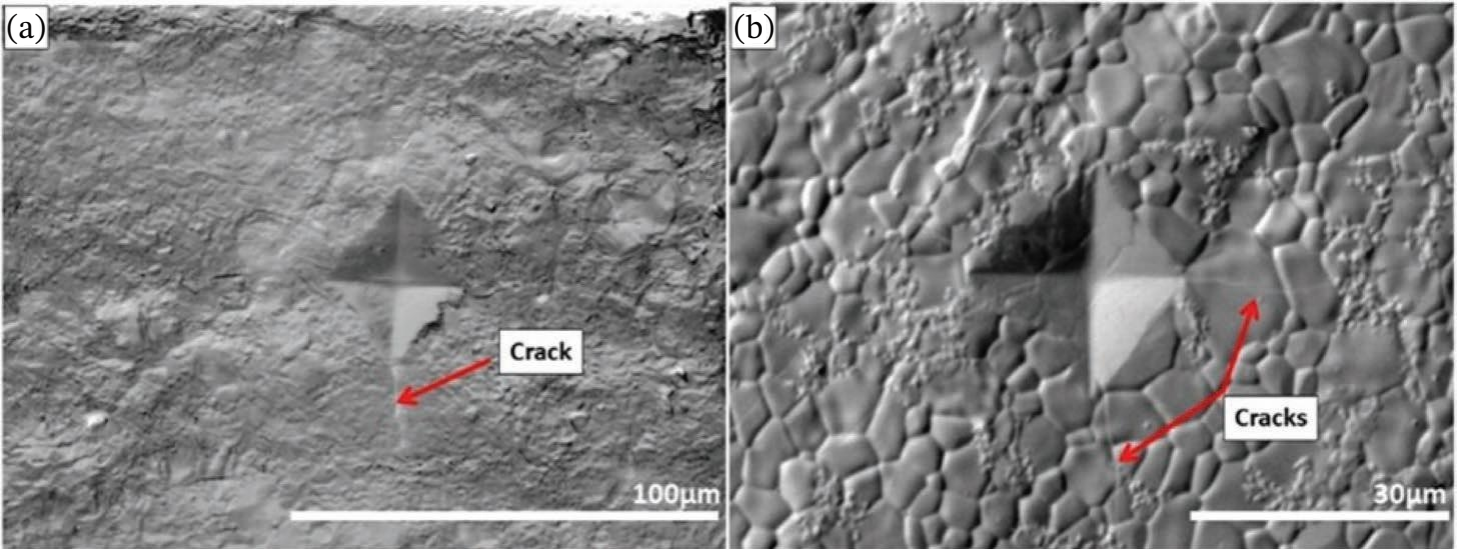
Images of cracks obtained by scanning electron microscopy after the Vickers microhardness test for (a) synthetic hydroxyapatite (HAp) and (b) synthetic hydroxyapatite–silver (HAp‐Ag).

The fracture cracks of the sintered samples resulting from the Vickers microhardness test were analyzed by SEM and are illustrated in Figure 5. Furthermore, Table 4 presents the results for fracture toughness, compressive strength, relative density, and porosity of the scaffolds. The results show an increase in fracture toughness of HAp‐Ag compared to HAp (0.94 and 0.66 MPa.m^1/2^, respectively). However, the compressive strength test showed that the addition of silver reduced the mechanical strength of the scaffolds (from 43.17 to 23.99 MPa).

**Table 4:** Results of fracture toughness, compressive strength, relative density, and porosity tests.

Formulation	Fracture toughness (MPa.m^1/2^)	Compressive strength (MPa)	Relative density (%)	Porosity (%)
HAp	0.66	43.17	74.36	16.43
HAp‐Ag	0.94	23.99	67.08	25.95

**Figure 6:**
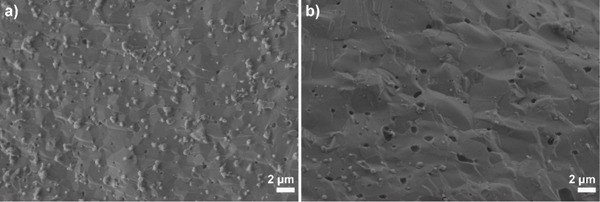
Cross‐sectional images of the pellets obtained by scanning electron microscopy after the compression strength test for (a) synthetic hydroxyapatite (HAp) and (b) synthetic hydroxyapatite–silver (HAp‐Ag).

Micrographs of the cross‐section of the pellets after the compression strength test are shown in Figure 6. The images show that both pure HAp and HAp‐Ag exhibit a microstructure typical of ceramics densified by high‐temperature sintering. A compact surface is observed, with coalesced grains and well‐defined grain boundaries. Porosity is observed in both samples, mainly in the form of isolated pores. Higher porosity is observed in HAp‐Ag compared to pure HAp.

**Figure 7:**
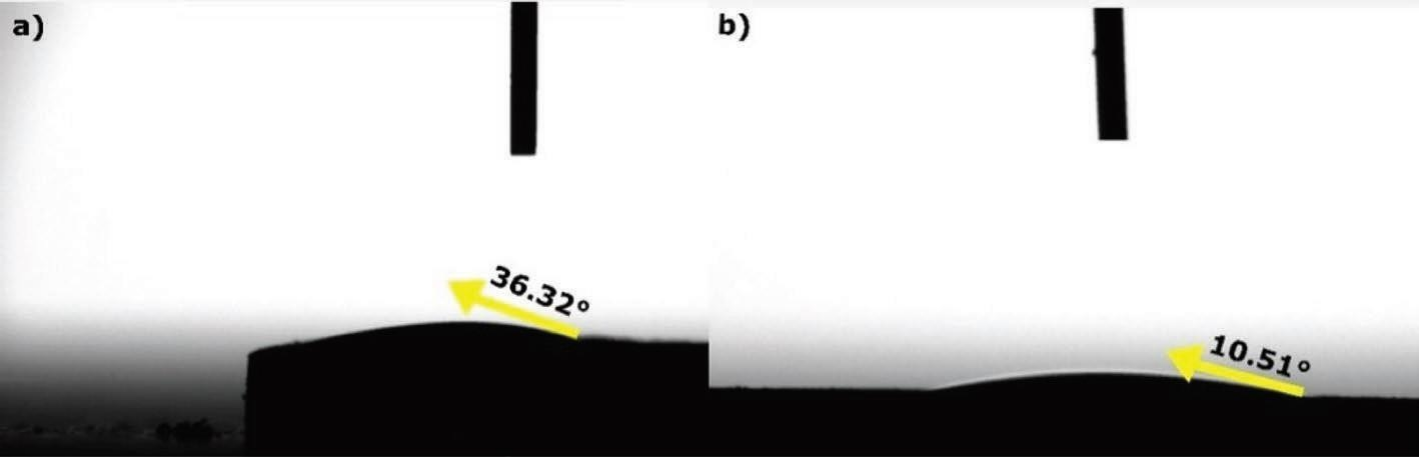
Contact angle measurements of biomaterials: (a) hydroxyapatite (HAp) and (b) synthetic hydroxyapatite–silver (HAp‐Ag).

Figure 7 shows the contact angle images of sintered HAp and Ag‐doped HAp biomaterials. For the pure HAp samples (Figure 7a), the average contact angle was 36.32°. On the other hand, Ag incorporation into HAp (Figure 7b) resulted in a more hydrophilic surface, with an average contact angle of 10.51°.

### 3.3. Cell Viability

**Figure 8:**
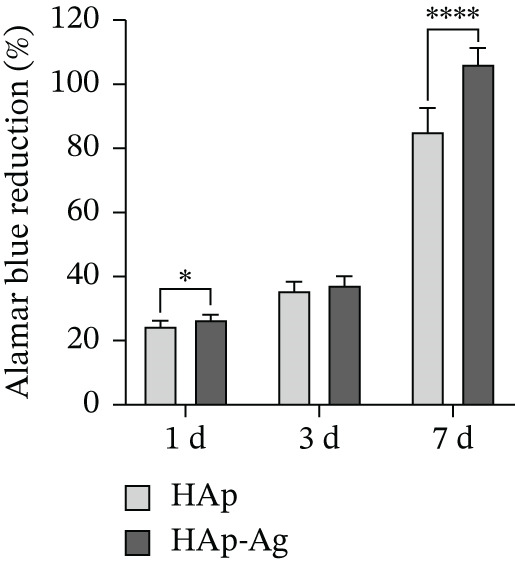
Metabolic activity of MC3T3‐E1 cells cultured on HAp and HAp‐Ag discs as assessed by the Alamar Blue reduction assay. Results are expressed as the percentage of Alamar Blue reduction (%). Data represent mean and standard deviation (*n* = 8). A statistically significant increase was observed for the HAp‐Ag group compared to HAp at Days 1 ( ^∗^ *p* < 0.05) and 7 ( ^∗∗∗∗^ *p* < 0.0001). Statistical comparisons were performed using unpaired *t*‐tests. CG, control group; HAp, hydroxyapatite; HAp‐Ag, hydroxyapatite–silver.

Alamar Blue reduction assay results demonstrated that MC3T3‐E1 preosteoblastic metabolic activity was higher on the surface of HAp‐Ag discs compared to HAp discs at all three time points (Figure 8 and Figure S1). Statistically significant differences were observed at Day 1 (*p* < 0.05) and Day 7 (*p* < 0.0001).

### 3.4. *μ*‐CT

**Figure 9:**
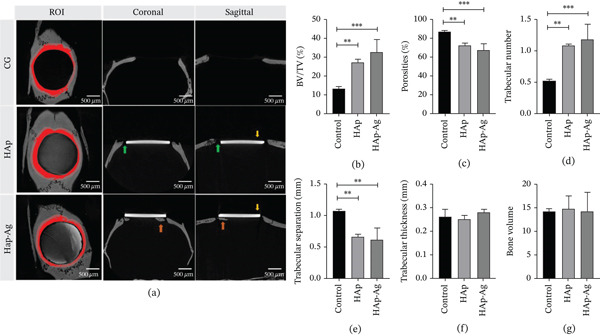
Analysis of the defect region by computed microtomography. (a) ROI delineation, coronal and sagittal views of the defect for the control group (CG), the synthetic hydroxyapatite (HAp) group, and the hydroxyapatite–silver (HAp‐Ag) group. The graphs represent the individual values and means with respective standard deviation of the following volumetric bone parameters: (b) bone fraction, (c) porosity, (d) number of trabeculae, (e) trabecular separation, (f) trabecular thickness, and (g) bone volume. Yellow arrowheads: biomaterial. Orange arrowheads: new bone. Green arrowheads: fibrous tissue. ANOVA followed by Tukey′s posttest.  ^∗^ *p* < 0.05,  ^∗∗^ *p* < 0.01, and  ^∗∗∗^ *p* < 0.001. *n* = 4 samples/group. CG, control group; HAp, hydroxyapatite; HAp‐Ag, hydroxyapatite–silver.

*μ*‐CT analysis (Figure 9) revealed a higher proportion of BV in relation to the total TV, a lower percentage of porosities, and a higher number of bone trabeculae in animals treated with HAp (*p* < 0.01) and HAp‐Ag (*p* < 0.001). Furthermore, the bone formed in the defect region of these animals presented a lower trabecular separation when compared to the CG (*p* < 0.05). No statistically significant differences were observed between the groups regarding trabecular thickness.

### 3.5. Histological Analysis (H&E)

**Figure 10:**
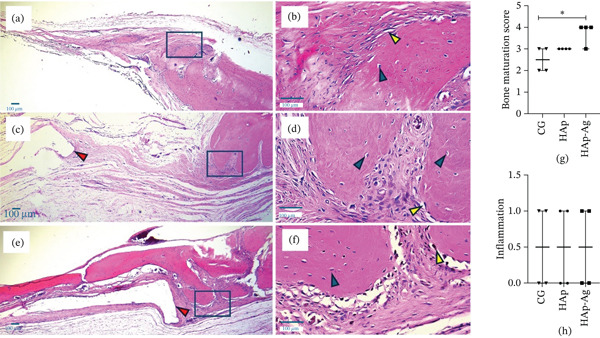
Histological analysis of a critical defect in rat calvaria. Low‐magnification images (×40, left column) showing the defect margin, which was outlined by blue rectangles to detail the newly formed bone (×400, right column). (a) Thin layer of fibrous connective tissue filling the defect area of the control group with the formation of island‐like bony structures and projections at the interface of the native bone edge. (b) Presence of osteocytes within the new bone and focal areas with flattened osteoblasts at the periphery. (c) Discrete formation of bony projections at the defect edge in HAp tissue. (d) Osteocytes and osteoblasts in the new bone. (e) HAp‐Ag tissue showing substantial formation of bony trabeculae interconnected with the native bone and in close proximity to the biomaterial area, extending to the central zone of the defect. (f) Numerous osteocytes and osteoblastic paving in the new bone. Red arrowheads: area occupied by the biomaterial at the end of the experimental period. Yellow arrowheads: osteoblasts. Blue arrowheads: osteocytes. Graphs of individual values, median, and 95% confidence interval illustrating scores of (g) bone maturation stage and (h) inflammation. The Kruskal–Wallis test followed by the Mann–Whitney posttest.  ^∗^ *p* < 0.05. H&E staining, original magnification: (a, c, e) ×40 and (b, d, f) ×400. *n* = 4 samples/group. CG, control group; HAp, hydroxyapatite; HAp‐Ag, hydroxyapatite–silver.

The CG (Figure 10a,b) and HAp group (Figure 10c,d) presented discrete bone formation, restricted to the defect edge, while the central area of the defect was filled mainly by loose connective tissue associated with the scattered presence of mononuclear inflammatory cells. Animals that received the silver‐containing biomaterial (HAp‐Ag) exhibited marked new bone formation, including mature bone structures, in continuity with the defect edge, consistent with the results of *μ*‐CT imaging (Figure 10e,f). Compared to the control, the HAp‐Ag group presented significantly higher bone maturation scores (*p* < 0.05; Figure 10g). In general, inflammatory infiltration was mostly absent or discrete in all groups (*p* > 0.05; Figure 10h).

### 3.6. Proinflammatory Cytokine Analysis (TNF‐*α*, IL‐6, and IL‐1*β*)

**Figure 11:**
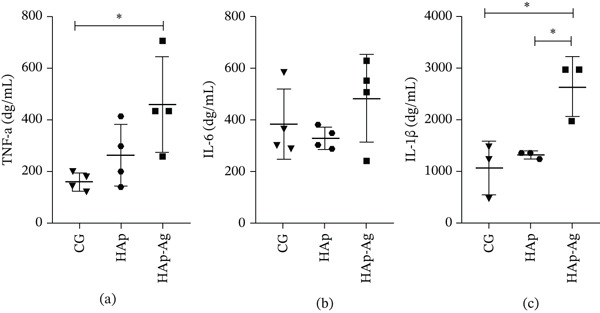
Graphs represent individual values and means with respective standard deviation for each study group regarding the dosage of proinflammatory tissue cytokines. (a) Tumor necrosis factor‐*α* (TNF‐*α*), (b) Interleukin 6 (IL‐6), and (c) Interleukin 1*β* (IL‐1*β*). ANOVA followed by Tukey′s posttest.  ^∗^ *p* < 0.05. *n* = 4 samples/group. CG, control group; HAp, hydroxyapatite; HAp‐Ag, hydroxyapatite–silver.

Quantification of proinflammatory cytokines by ELISA (Figure 11) demonstrated that animals in the HAp‐Ag group had higher levels of TNF‐*α* compared to animals that did not receive biomaterial (*p* < 0.05) and IL‐1*β* when compared to the other study groups (*p* < 0.05). Tissue levels of IL‐6 did not differ between groups.

### 3.7. RT‐qPCR

**Figure 12:**
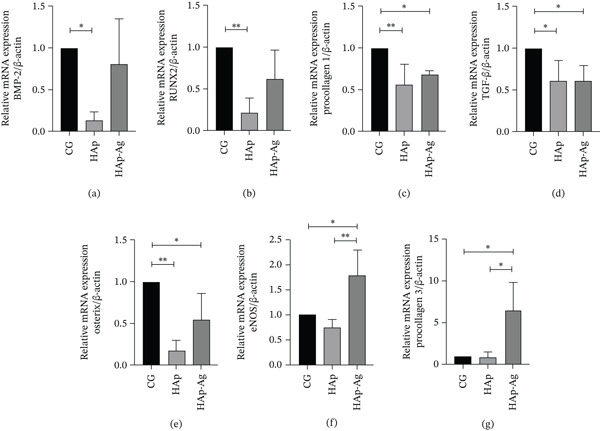
Bar graphs represent the means and standard deviation of each group regarding the relative mRNA expression of (a) Bone Morphogenetic Protein 2 (BMP‐2), (b) Runt‐Related Transcription Factor 2 (RUNX2), (c) Procollagen 1, (d) transforming growth factor *β* (TGF‐*β*), (e) Osterix, (f) endothelial nitric oxide synthase (eNOS), and (g) Procollagen 3. Reference gene: *β*‐actin. ANOVA followed by Tukey′s posttest.  ^∗^ *p* < 0.05 and  ^∗∗^ *p* < 0.01. *n* = 4 samples/group. CG, control group; HAp, hydroxyapatite; HAp‐Ag, hydroxyapatite–silver.

RT‐qPCR analysis demonstrated that the tissue surrounding the critical defect of animals with HAp‐Ag had higher relative expression of eNOS and Procollagen 3 mRNA compared to the CG (*p* < 0.05) and HAp (*p* < 0.01 and *p* < 0.05, respectively). For the markers BMP‐2, RUNX2, Procollagen 1, TGF‐*β*, and Osterix, there was no statistically significant difference between the groups treated with biomaterial. The negative CG showed higher relative expression of mRNA, compared to the HAp group, for all these markers, and compared to the HAp‐Ag group, for Procollagen 1, TGF‐*β*, and Osterix (Figure 12).

## 4. Discussion

The present study characterized the synthesis of a new biomaterial based on synthetic HAp and silver and investigated its cytotoxicity and biological effect for bone regeneration in an experimental model of critical defect in rat calvaria. The null hypothesis, which considered that the incorporation of silver into HAp does not alter the physical properties and biological potential of new bone formation in a critical‐size bone defect, was rejected. Silver doping to HAp provided a biomaterial with greater fracture toughness and more hydrophilicity, as well as with a greater potential to promote cell viability and induce bone maturation.

HAp is a compound widely studied for use in orthopedic and dental implants due to its chemical composition similar to that of the mineralized tissues of the human body, in addition to presenting excellent bioactivity [18, 37]. Several studies have explored ionic substitution or doping of HAp as a strategy to improve its physicochemical and osteogenic properties, with silver being a prominent element due to its promising effects in this context [38, 39].

XRD analysis revealed that the incorporation of silver into HAp did not lead to the formation of a new detectable crystalline phase (i.e., the majority phase in the HAp‐Ag samples is crystalline HAp). The absence of a new phase, in this case, may be due to the low concentration of silver in the sample, which is outside the sensitivity limit of the XRD instrument, or to the incorporation of the ion into the crystalline structure through the ionic substitution of silver by calcium [20]. Furthermore, the lower intensity of the peaks in the HAp‐Ag samples indicates interferences in the HAp crystal lattice. These, in turn, may be related to the difference in the size of the ionic radius of Ag (1.28 Å) compared to Ca (0.99 Å), reinforcing the occurrence of ionic substitution, since the packing of larger atoms tends to distort the lattice parameters [40–43].

The elemental chemical analysis by XRF of the powders showed that, for HAp‐Ag, the percentage of silver was very close to the theoretical value (0.1%). Regarding the (Ca + Ag)/P ratio, both samples presented values below the theoretical HAp (1.67). According to Narasaraju and Phebe [44], this is common in calcium phosphates prepared by wet methods such as coprecipitation. Furthermore, this stoichiometric deviation is favorable to the material obtained for two reasons: (1) Bone HAp has a Ca/P ratio between 1.5 and 1.85 [45], and (2) the apatite structure formed with a Ca/P ratio lower than 1.67 exhibits high solubility and enhanced bioactivity [46].

FT‐IR spectroscopy was employed to investigate the functional groups present in HAp and HAp‐Ag. In both samples, an intense band was observed at ~1639 cm^−1^ and two bands between 3400 and 3600 cm^−1^ attributed to adsorbed water (H‐O‐H bending mode) [47, 48]. The characteristic bands of the PO_4_^3−^ ion were identified in different regions of the spectrum, including ~470 cm^−1^ (*ν*_2_), 560–605 cm^−1^ (*ν*_4_), ~963 cm^−1^ (*ν*_1_), and ~1035 cm^−1^ (*ν*_3_), confirming the formation of the apatite structure [49]. The band at ~877 cm^−1^ corresponds to HPO_4_^2−^ ions, and the peaks between 1400 and 1500 cm^−1^ are attributed to CO_3_^2−^ groups adsorbed during coprecipitation synthesis [50, 51]. Furthermore, no additional bands were observed in HAp‐Ag, indicating the absence of impurities or secondary phases due to doping. However, small variations in the intensity and width of the bands, especially those associated with water and phosphate groups, are indicative of the incorporation of the dopant into the HAp structure [48].

Regarding the mechanical properties, pure HAp pellets showed a fracture toughness of 0.66 MPa.m^1/2^, and silver incorporation enhanced this value by roughly 42% (0.94 MPa.m^1/2^). However, the compressive strength test showed that the addition of silver reduced the mechanical strength of the pellets. This contrast can be explained by the relative density and porosity of the scaffolds. During sintering, pores are formed in the HAp structure, thus reducing the densification of the material [34]. In bioceramics such as HAp, the presence of pores naturally reduces their mechanical strength. However, the same does not happen with fracture toughness, since the pores can act as energy dissipation mechanisms and deflect crack propagation, hindering the rapid advancement of fracture [52, 53]. Thus, although the material becomes less resistant in terms of maximum supported load, it may exhibit a greater capacity to absorb energy before fracture. Zhang et al. [54], in a study that evaluated the changes in mechanical properties by the addition of silver oxide to HAp, found fracture toughness values of 0.70 MPa.m^1/2^ for HAp and 0.95 MPa.m^1/2^ for HAp‐Ag, with an improvement in this property concomitant with the increase in silver concentration in the sample. This toughening mechanism is also attributed to the fact that silver particles serve as obstacles to crack propagation, interrupting or diverting cracks, in addition to acting as bridges between the two ends of the cracks through elastic deformation, delaying their complete opening [54]. As a consequence, more energy is required for the crack to continue propagating in the composite, resulting in greater fracture toughness. Despite this, the values obtained in our study for HAp‐Ag samples are still lower than those of cortical bone, which range from 2 to 6 MPa.m^1/2^[55].

Heat treatment at 1200°C for 2 h tends to promote a high degree of densification and grain growth in HAp [56, 57]. This reduces the amount of open porosity and the interconnectivity of pores, leading to a predominantly dense microstructure. Indeed, in both samples, porosity was observed mainly in the form of isolated pores. In the case of the HAp‐Ag sample, the microstructure exhibited a morphology similar to that of pure HAp, suggesting that silver incorporation did not significantly alter the densification mechanism or grain growth of HAp. However, a higher number of pores was evident in HAp‐Ag compared to pure HAp, which is consistent with the results of relative density, porosity, and mechanical properties. Regarding the application of granules in bone regeneration, dense bioceramics are likely to exhibit reduced osteoconduction, as bone integration is favored by the presence of open and interconnected porosity, typically in the range of tens to hundreds of micrometers [58]. Since the pores observed in the micrographs are mostly micrometric and isolated, it is plausible that the granule structure is less favorable for deep cell colonization and internal bone ingrowth, which may reduce, for example, its resorption capacity.

In addition to mechanical properties, another fundamental aspect to be considered in the development of biomaterials for bone regeneration is hydrophilicity, that is, the tendency of a liquid to spread over its solid surface [59]. The more hydrophilic the biomaterial, the greater its interaction with tissue fluids tends to be and, consequently, the greater the level of protein interaction, adhesion, and cell proliferation [60, 61]. Our results demonstrated that the addition of silver to HAp promoted a reduction in the contact angle, which indicates an increase in hydrophilicity. Similarly, Hu and Zhou [61], when evaluating the effect of incorporating different concentrations of silver nanoparticles into porous scaffolds of *β*‐tricalcium phosphate and gelatin, found that the higher the silver concentration, the lower the contact angle in the wettability test. In this sense, this effect may contribute positively to the biological potential of HAp‐Ag samples, favoring tissue repair and integration with bone tissue.

After the physical evaluation of the biomaterials and confirmation that the pellets promoted changes in physical properties due to the incorporation of silver, we conducted an *in vivo* evaluation to analyze their performance in critical‐size bone defects. The experimental calvarial defect model is widely used in studies investigating the bone regeneration potential of biomaterials [62], and the term “critical‐size defect” refers to the smallest size of the bone wound that will not heal spontaneously over the experimental period [63, 64]. An 8‐mm bone defect in the rat calvaria is considered critical, while defects of smaller diameters can result in considerable spontaneous bone growth, especially after 60 days [65].

Volumetric analysis by *μ*‐CT revealed a higher bone fraction and better bone quality parameters (porosity, number of trabeculae, and trabecular separation) in animals treated with biomaterials. These findings complement the results of the cell viability assay, which showed higher MC3T3‐E1 preosteoblastic viability on HAp‐Ag discs, and the histological analysis, which revealed greater bone maturation in the HAp‐Ag group compared to the control, with no significant differences between control and HAp or between HAp and HAp‐Ag. Taken together, these results suggest that the incorporation of silver into HAp also provided a slight improvement in its osteogenic properties.

In a study carried out by García‐Ortiz et al. [38] that evaluated a HAp‐Ag composite obtained by the precipitation method, it was found that the addition of AgNO_3_ to HAp increased the mineralization of osteoblast‐like cells of the MG‐63 lineage after 14 days of experiment. During the mineralization stage, proteins such as osteocalcin, osteopontin, and alkaline phosphatase play an important role in favoring the binding of calcium and phosphate to the nonmineralized bone matrix [66–68]. The literature [69, 70] reports that when ions with a lower positive charge than calcium, such as silver, are doped in HAp, there is an increase in the negative zeta potential, which reduces the electrostatic repulsion between the material and the proteins in the medium (which are negatively charged at physiological pH). In addition, obtaining a more hydrophilic surface also enhances protein interaction, adhesion, and cell proliferation, as mentioned previously. These mechanisms, in turn, support the justification that a silver‐doped biomaterial has greater potential for bone formation and mineralization.

Regarding tissue cytokine levels, our study found that the use of pure HAp pellets did not induce an increase in the proinflammatory markers analyzed. This finding is in agreement with the study by Carvalho Vasconcelos et al. [31], which evaluated the effects of various biomaterials in an experimental model of critical‐size calvarial defects at 90 days and found similar levels of IL‐1*β* and TNF‐*α* between animals with untreated and HAp‐treated bone defects. In contrast, higher levels of these cytokines were observed in animals from the HAp‐Ag group. Chronic inflammation is detrimental to bone consolidation by favoring the differentiation and activation of osteoclasts and suppressing bone formation by osteoblasts [71]. This may have limited the bone regeneration process mediated by the silver‐containing biomaterial. It is noteworthy to mention, however, that histological analysis of tissue inflammation did not show statistically significant differences between the groups, which may suggest that, at a macrolevel, the increase in proinflammatory cytokines did not reflect an increase in inflammatory cells in the defect region.

RT‐qPCR data demonstrated that the HAp‐Ag biomaterial promoted an increase in the relative gene expression of eNOS and Procollagen 3. eNOS corresponds to the endothelial isoform of the enzyme responsible for the synthesis of nitric oxide, and its action is essential for the induction of angiogenesis and cell permeability [72]. Procollagen 3 is equivalent to the precursor of Type 3 collagen, whose expression is increased during the initial phase of the healing process of various tissues, including bone [73]. A higher expression of these two markers in the soft tissue adjacent to the bone defect region may indicate a maintenance of the silver stimulus to revascularization, even after 90 days of tissue injury. Although eNOS and Type 3 collagen have been shown to play a role in regulating osteoblast activity [73, 74], their increased gene expression cannot be associated with equivalent persistence of bone repair, since the expression of osteoblastic differentiation markers and bone neoformation did not accompany this increase. The expression of Osterix, observed in the present study, represents an essential milestone in osteoblast differentiation. It is a fundamental transcription factor, whose activation occurs hierarchically after the induction of RUNX2, and is essential for osteoprogenitor cells to complete their maturation process and acquire the functional capacity to synthesize and mineralize bone matrix [75]. Previous studies demonstrate that the absence of OSX prevents bone formation, even in the presence of RUNX2 [76], which reinforces its central role in intramembranous ossification, especially in craniofacial bones such as the calvaria. In the present experimental model, no greater gene expression of OSX was observed in animals treated with the HAp‐Ag composite, compared to the CG. Taken together, the results of the molecular analyses indicate that the presence of silver does not appear to favor bone formation over a prolonged evaluation period.

The fact that the molecular analyses correspond to a temporal and momentary cut‐off of the 90 days of healing may justify the inconsistency between the results of the cellular and morphological analyses (histology and *μ*‐CT), which evaluated the early and cumulative effects of the treatment on the bone tissue throughout the entire experimental period, respectively. Future studies should focus on understanding the molecular events in earlier periods of healing.

Although the results presented are promising, it is important to point out that our work has limitations. Ag^+^ ion release analysis, which was not performed in this study, could have provided a better understanding of the differences observed between early and late healing periods. Histological and *μ*‐CT images demonstrated failures in the integration between the newly formed bone and the biomaterial. The main reason for this can be attributed to the method of obtaining the discs, based on the fact that compression for manufacturing the block graft generates a biomaterial with no or a minimal amount of pores, compromising cellular anchorage and blood circulation for supplying oxygen and nutrition, with a direct impact on osteoconduction [77]. On the other hand, this limitation can be partially overcome in clinical situations in which primary stability of the material is obtained, as occurs in the installation of dental implants [32]. In addition, complementary characterization tests, such as the degradability of the material, are relevant, considering that, after 90 days of observation, the tablets showed no signs of resorption. This behavior can compromise their performance, since, ideally, the biodegradation rate should be synchronized with the rate of bone neoformation [78, 79]. If the biomaterial persists for an excessive amount of time in the bone bed, it can act as a physical barrier to natural remodeling, hinder replacement by mature bone, and induce chronic inflammatory reactions or fibrous encapsulation.

Although HAp is a recognized resorbable biomaterial, with the capacity to be progressively replaced by newly formed bone tissue, silver, in turn, is not resorbed in the same way. In silver‐containing biomaterials, silver ions are expected to be gradually released into the local microenvironment, especially in the early stages after implantation, promoting a desirable antimicrobial effect [80]. However, some silver may remain in the tissue, either retained as inorganic precipitates, phagocytosed by immune cells, or sequestered in fibrous tissue [81, 82]. In the formulation used in this study, HAp was associated with a low concentration of silver (0.1% of AgNO₃), which aims to ensure a balance between antimicrobial efficacy and biocompatibility. Future investigations are recommended to monitor the long‐term biodistribution of silver, as well as its possible persistence in adjacent tissues, in order to ensure a full understanding of the systemic and local effects of this metallic component in translational clinical contexts.

Finally, it is important to highlight that the main motivation for incorporating silver into HAp was to improve its mechanical properties, which was confirmed by fracture toughness analysis. However, when evaluating the performance of the biomaterial in a critical calvarial defect model, the focus was to verify whether the addition of silver would compromise the biological properties of pure HAp. It is worth noting that the model used does not allow the evaluation of bone repair under mechanical loading conditions [62]; therefore, the biological outcomes obtained in this study should not be extrapolated to applications in areas subject to mechanical stress.

## 5. Conclusion

The findings of the present study indicate that silver incorporation into HAp modifies its physicochemical and mechanical properties, leading to increased fracture toughness and porosity, along with reduced compressive strength and enhanced wettability. Additionally, HAp‐Ag promoted higher preosteoblastic metabolic activity and improved in vivo bone formation, with evidence of more advanced bone maturation. The upregulation of TNF‐*α*, IL‐1*β*, Procollagen 3, and eNOS suggests an active role in modulating the bone healing microenvironment. Overall, despite the reduction in compressive strength, silver incorporation did not impair the biological performance of HAp and may enhance its potential for bone regeneration applications.

## Funding

The study was funded by the Conselho Nacional de Desenvolvimento Científico e Tecnológico, 10.13039/501100003593, 303915/2023‐4 and 401672/2023‐9, and the INCT iCEIS, 406264/2022‐8.

## Ethics Statement

The experimental protocol of this study was submitted to the Ethics Committee for Teaching and Research on Animals (CEUA)–Federal University of Rio Grande do Norte (UFRN) and approved under protocol number 019/2020.

## Conflicts of Interest

The authors declare no conflicts of interest.

## Supporting Information

Additional supporting information can be found online in the Supporting Information section.

## Supporting information


**Supporting Information 1** Table S1: Forward and reverse primer sequences. Footnote: BMP‐2, Bone Morphogenetic Protein 2; RUX2, Runt‐Related Transcription Factor 2; TGF‐*β*, transforming growth factor *β*; eNOS, endothelial nitric oxide synthase.

**Supporting Information 2** Figure S1: Metabolic activity of MC3T3‐E1 cells cultured on HAp and HAp‐Ag discs, assessed by Alamar Blue reduction assay. The values obtained at Days 1, 3, and 7 were normalized to those of the control condition (cells cultured on standard tissue culture polystyrene), which was set at 100% (dashed line) at each time point. Data are shown as mean ± standard deviation (SD). A significant increase was observed in the HAp‐Ag group compared to the HAp group at Day 1 (*p* < 0.05) and Day 7 ( ^∗∗∗^ *p* < 0.0001). Statistical comparisons were performed using unpaired *t*‐tests. CG, control group; HAp, hydroxyapatite; HAp‐Ag, hydroxyapatite–silver.

## Data Availability

The experimental data reported in this work are available upon reasonable request by e‐mail to the corresponding author.
