# Supplementary material for: Physicochemical Characterization, Cytotoxicity, and In Vivo Evaluation of a Hydroxyapatite–Silver Composite for Bone Regeneration
Source: Biomed Res Int. 2026 Jun 4;2026:8065442. doi: 10.1155/bmri/8065442 (PMC13238255; doi:10.1155/bmri/8065442)
Supplement: Supplementary file 1 — Supporting Information 1 Table S1: Forward and reverse primer sequences. Footnote: BMP‐2, Bone Morphogenetic Protein 2; RUX2, Runt‐Related Transcription Factor 2; TGF‐β, transforming growth factor β; eNOS, endothelial nitric oxide synthase. [file BMRI-2026-8065442-s001.docx]

**Table S1** Forward and Reverse primer sequences

| **Primer** | **Sequence** | |
| --- | --- | --- |
| β-actin | Foward | AGGCCAACCTGTAAAAGATG |
|  | Reverse | TGTGGTACGAGAGGCATAC |
| BMP-2 | Foward | GGGACCCGCTGTCTTCTAGT |
|  | Reverse | TCAACTCAAATTCGCTGAGGAC |
| RUNX-2 | Foward | CCTCTGACTTCTGCCTCTGG |
|  | Reverse | TAAAGGTGGCTGGGTAGTGC |
| Osterix | Forward | TCTCCATCTGCCTGACTCCT |
|  | Reverse | AGCGTATGGCTTCTTTGTGC |
| eNOS | Forward | TCCGGAAGGCGTTTGATC |
|  | Reverse | GCCAAATGTGCTGGTCACC |
| Procollagen 1 | Forward | CAGGGAGTAAGGGACACGAA |
|  | Reverse | TCCCACAGCAGTTAGGAACC |
| Procollagen 3 | Forward | AGTGTGGCTTTCAGT TCAGC |
|  | Reverse | TGGGGTTTCAGAGAGTTTGG |

BMP-2, Bone morphogenetic protein 2; RUX2, Runt-related transcription factor 2; TGF-β, Transforming growth factor β; eNOS, Endothelial nitric oxide synthase.

|  |
| --- |
|  |
