# Supplementary figures and images for: Physicochemical Characterization, Cytotoxicity, and In Vivo Evaluation of a Hydroxyapatite–Silver Composite for Bone Regeneration
Source: Biomed Res Int. 2026 Jun 4;2026:8065442. doi: 10.1155/bmri/8065442 (PMC13238255; doi:10.1155/bmri/8065442)

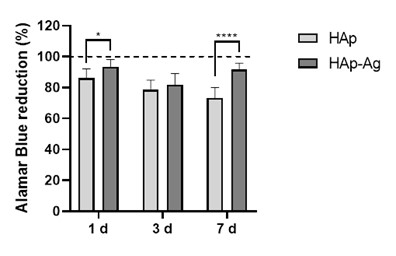

Supplement: Supplementary file 2 — Supporting Information 2 Figure S1: Metabolic activity of MC3T3‐E1 cells cultured on HAp and HAp‐Ag discs, assessed by Alamar Blue reduction assay. The values obtained at Days 1, 3, and 7 were normalized to those of the control condition (cells cultured on standard tissue culture polystyrene), which was set at 100% (dashed line) at each time point. Data are shown as mean ± standard deviation (SD). A significant increase was observed in the HAp‐Ag group compared to the HAp group at Day 1 (p < 0.05) and Day 7 ( ∗∗∗ p < 0.0001). Statistical comparisons were performed using unpaired t‐tests. CG, control group; HAp, hydroxyapatite; HAp‐Ag, hydroxyapatite–silver. [file BMRI-2026-8065442-s002.jpg]
